# Strategies to Avoid Wrongly Labelled Genomes Using as Example the Detected Wrong Taxonomic Affiliation for *Aeromonas* Genomes in the GenBank Database

**DOI:** 10.1371/journal.pone.0115813

**Published:** 2015-01-21

**Authors:** Roxana Beaz-Hidalgo, Mohammad J. Hossain, Mark R. Liles, Maria-Jose Figueras

**Affiliations:** 1 Unitat de Microbiologia, Departament de Ciènces Médiques Bàsiques, Facultat de Medicina i Ciències de la Salut, IISPV, Universitat Rovira i Virgili, Reus, Spain; 2 Department of Biological Sciences, Auburn University, Auburn, Alabama, United States of America; Cankiri Karatekin University, TURKEY

## Abstract

Around 27,000 prokaryote genomes are presently deposited in the Genome database of GenBank at the National Center for Biotechnology Information (NCBI) and this number is exponentially growing. However, it is not known how many of these genomes correspond correctly to their designated taxon. The taxonomic affiliation of 44 *Aeromonas* genomes (only five of these are type strains) deposited at the NCBI was determined by a multilocus phylogenetic analysis (MLPA) and by pairwise average nucleotide identity (ANI). Discordant results in relation to taxa assignation were found for 14 (35.9%) of the 39 non-type strain genomes on the basis of both the MLPA and ANI results. Data presented in this study also demonstrated that if the genome of the type strain is not available, a genome of the same species correctly identified can be used as a reference for ANI calculations. Of the three ANI calculating tools compared (ANI calculator, EzGenome and JSpecies), EzGenome and JSpecies provided very similar results. However, the ANI calculator provided higher intra- and inter-species values than the other two tools (differences within the ranges 0.06–0.82% and 0.92–3.38%, respectively). Nevertheless each of these tools produced the same species classification for the studied *Aeromonas* genomes. To avoid possible misinterpretations with the ANI calculator, particularly when values are at the borderline of the 95% cutoff, one of the other calculation tools (EzGenome or JSpecies) should be used in combination. It is recommended that once a genome sequence is obtained the correct taxonomic affiliation is verified using ANI or a MLPA before it is submitted to the NCBI and that researchers should amend the existing taxonomic errors present in databases.

## Introduction

Members of the genus *Aeromonas* are found in aquatic environments worldwide and have been implicated in human and fish diseases [3, 12]. The genus now accounts for 27 species and since the year 2008 ten new species have been discovered mainly thanks to the use of housekeeping genes such as *rpoD* and *gyrB* [8, 19]. Housekeeping genes evolve faster than the 16S rRNA, have a higher resolution for differentiating closely related species and therefore are more reliable for the correct identification of *Aeromonas* strains to species level [8]. The multilocus phylogenetic analysis (MLPA) or multilocus sequence analysis (MLSA) of at least five concatenated housekeeping genes is an accurate tool for the delineation of *Aeromonas* species [8, 20]. The MLPA increases taxonomic resolution and makes more robust the overall phylogenetic relatedness between taxa [8]. This analysis is therefore a straight forward alternative to substitute the complex and time-consuming DNA-DNA hybridization (DDH) method for establishing species identity [8, 18, 20, 21, 24, 25, 27]. The correlation found between DDH results >70% and values of 95–96% for the *in silico* determined average nucleotide identity (ANI) between two genomes lead to the proposal to use ANI as the “gold standard” to substitute DDH for species delineation [5, 9, 15, 16, 17, 22]. Therefore, ANI is a similarity index between a given pair of genomes that determines if they belong or not to the same species (using a minimum of 20% of their genomes) [22]. There are two ANI calculation systems, the ANIm performed using the MUMer rapid alignment system [22] and the ANIb that uses BLAST [9]. The ANIb is the approach more widely used for bacterial identification [5]. The most commonly used tools to calculate ANI are available at 3 web-sites. The first available, named JSpecies, was developed by Richter and Rosselló-Móra [22] to determine if two genomes belong or not to the same species. It provides a software tool for calculating both ANIb and ANIm simultaneously and enables multiple ANI calculations between different genomes. The latter is in contrast with the other two web-based tools in which only ANIb can be measured and each online analysis is run separately for each pair of genomes. These two web-based tools are the ANI calculator, offered by the Environmental Microbial Genomics Laboratory at the Georgia Institute of Technology developed by the team of Konstantinidis [23] and the one at the curated EzGenome database (at the EZBIOCLAUD) developed by Chun from the Seoul National University, Republic of Korea. The objective of the latter is to amend taxonomic errors at the NCBI, identifying manually the genomes using ANI and the 16S rRNA sequences and comparing them with the EzTaxon database [14].

Within an ongoing study that aimed to determine the taxonomic status (at the level of subspecies) of a group of *A. hydrophila* isolates responsible for an epidemic catfish disease outbreak in Alabama, United States [11], the genome of a representative strain (ML09-119) was compared using ANI with the available *A. hydrophila* genomes at GenBank, obtaining values > 95% with several genomes (including the type strain of the species *A. hydrophila* ATCC 7966^T^). However, with other *A. hydrophila* genomes the ANI was far below the species boundary (95%) indicating that these genomes did not affiliate with *A. hydrophila* and were therefore wrongly named. Considering the relevance of this observation and the fact that a correct identification of *Aeromonas* spp. is difficult, the aim of this study was to re-evaluate the species identity of the 44 available genomes (up to July 2014) of this genus in order to detect those that have been misidentified and to give recommendations of how to avoid this problem in the future using the available molecular tools.

## Materials and Methods

We studied the 44 existing annotated *Aeromonas* genomes (up to 23 of July 2014) in the GenBank Genome database of the NCBI (http://www.ncbi.nlm.nih.gov/genome/?term=Aeromonas) of which only five belonged to type strains (Table 1). Of these, 20 were labeled as belonging to *A. hydrophila* (including the type strain ATCC 7966^T^), seven as *A. veronii*, five as *A. salmonicida* subspecies (with one type strain of the subsp. *pectinolytica* 34mel^T^), two as *A. caviae*, two as *Aeromonas* sp., one as both species *A. hydrophila/A. dhakensis* and one of each of the species *A. dhakensis, A. media, A. jandaei, A. enteropelogenes* (synonym of *A. trota*), *A. diversa* (CDC 2478-85^T^), *A. molluscorum* (848^T^ = CECT 5864^T^) and *A. taiwanensis* (LMG 24683^T^). The 44 genomes were re-identified using the MLPA proposed by Martínez-Murcia et al. [19] with the sequences of six housekeeping genes (*gyrB, rpoD, recA, dnaJ, gyrA* and *dnaX*) and performing ANI calculations. The genes were retrieved from each individual genome and were aligned with those of an in house database used to define recently published species and that contained those genes for all the type strains of the known *Aeromonas* spp. [1, 19]. In three genomes (*Aeromonas* sp. 159, *A. enteropelogenes* 1991CR and *A. salmonicida* subsp. *salmonicida* 34mel^T^) some genes were missing, presumably due to lower genome coverage, and the analysis was performed with four or five available genes. Genetic distances and clustering of the concatenated sequences were obtained using Kimura’s two-parameter model and phylogenetic trees were constructed with the neighbor-joining method using the MEGA software version 6 [26]. The web-based service ANI calculator (http://enve-omics.ce.gatech.edu/ani/index) was used for comparing the 44 *Aeromonas* genomes against the one of *A. hydrophila* (ML09-119) used as an inter-species reference. For the intra-species ANI calculation, genomes were compared with the type strain when available or with a selected genome of the same species.

**Table 1:** Intra and inter-species ANI values for the 44 *Aeromonas* genomes and identification on the basis of the MLPA and ANI (mislabeled genomes in bold).

		**ANI values with Identification**		
**Reference at NCBI**	**Origin (Country)**	***A. hydrophila* ML09-119**	**Representative genomes**	**MLPA and ANIb**
			**ATCC7966^T^**	
*A. hydrophila* subsp. *hydrophila* ATCC 7966^T^ ^a^	Canned milk (US)	97.14	100	*A. hydrophila*
*A. hydrophila* ML09-119^a^	Diseased outbreak in catfish	100	97.14	*A. hydrophila*
*A. hydrophila* AL09-71	Diseased catfish (USA)	99.99	97.24	*A. hydrophila*
*A. hydrophila* pc104A	Soil of catfish pond (USA)	99.99	97.30	*A. hydrophila*
*A. hydrophila* 226	Clinical strain (Malaysia)	97.13	97.30	*A. hydrophila*
*A. hydrophila* AE34	Kidney of a moribund carp (Japan)	97.20	97.22	*A. hydrophila*
*A. hydrophila* SNUFPC-A8^a^	Kidney of moribund salmon (Korea)	97.11	97.10	*A. hydrophila*
*A. hydrophila* AD9	Wetland sediments (USA)	97.13	97.16	*A. hydrophila*
*A. hydrophila* NF2	Necroziting fascitiis (USA)	96.94	97.29	*A. hydrophila*
*A. hydrophila* NF1	Necroziting fascitiis (USA)	96.79	97.15	*A. hydrophila*
			**SSU**	
***A. hydrophila/A. dhakensis* SSU^a^**	**Stool (USA)**	**93.75**	**100**	***A. dhakensis***
***A. hydrophila* 113^a^**	**Clinical strain (Malaysia)**	**93.74**	**97.57**	***A. dhakensis***
***A. hydrophila* 187^a^**	**Clinical strain (Malaysia)**	**93.70**	**97.60**	***A. dhakensis***
***A. hydrophila* 173^a^**	**Clinical strain (Malaysia)**	**93.72**	**97.54**	***A. dhakensis***
***A. hydrophila* 259^a^**	**Clinical strain (Malaysia)**	**93.70**	**97.56**	***A. dhakensis***
***A. hydrophila* 277^a^**	**Clinical strain (Malaysia)**	**93.76**	**97.53**	***A. dhakensis***
***A. hydrophila* 145**	**Clinical strain (Malaysia)**	**93.84**	**97.48**	***A. dhakensis***
***A. hydrophila* 14^a^**	**Clinical strain (Malaysia)**	**93.77**	**97.54**	***A. dhakensis***
***A. hydrophila* YL17**	**Compost (Malaysia)**	**93.76**	**97.34**	***A. dhakensis***
*A. dhakensis* AAK1^a^ ^,^ ^b^	Blood of a patient with septicemia and necrotizing fasciitis (Taiwan)	93.73	97.52	*A. dhakensis*
*Aeromonas* sp. MDS8^a^ ^,^ ^b^	Sludge of a dairy treatment plant (India)	93.71	97.57	*A. dhakensis*
			**B565**	
*A. veronii* B565^a^	Aquaculture pond sediment (China)	88.56	100	*A. veronii*
*A. veronii* AER39^a^	Human blood (USA)	88.38	96.76	*A. veronii*
*A. veronii* AER397^a^	Human blood (USA)	88.35	99.72	*A. veronii*
*A. veronii* AMC35^a^	Under eye wound (USA)	88.33	97.21	*A. veronii*
*A. veronii* Hm21^a^	Leech digestive tract (France)	88.47	96.83	*A. veronii*
*A. veronii* Phln2^a^	Fish intestine (India)	88.51	96.75	*A. veronii*
*Aeromonas* sp. 159^a^ ^,^ ^c^	Stool (Malaysia)	88.40	96.75	*A. veronii*
***A. veronii* AMC34^a^**	**Human faeces (USA)**	**88.54**	**94.68**	“***Aeromonas* sp.nov.”**
			**A449**	
*A. salm.* subsp. *salmonicida* A449^a^	Brown trout with furunculosis (France)	88.23	100	*A. salmonicida*
*A. salm.* subsp. *salmonicida* 01-B526^a^	Infected brook trout (Canada)	88.28	99.98	*A. salmonicida*
*A. salm.* subsp. *pectinolytica* 34mel^T^ ^a^	Heavily polluted river (Argentina)	88.33	97.40	*A. salmonicida*
*A. salm.* subsp. *masoucida* NBRC13784	Not specified (Japan)	88.35	99.82	*A. salmonicida*
*A. salm.* subsp. *achromogenes* AS03^a^	Ulcer from crucian carp (Korea)	88.57	99.79	*A. salmonicida*
			**Ae398**	
*A. caviae* Ae398^a^ ^,^ ^d^	Feces from a 17month old boy with gastroenteritis (Brazil)	89.00	100	*A. caviae*
*A. caviae* YL12	Compost (Malaysia)	89.02	98.27	*A. caviae*
***A. hydrophila* HZM**	**Tropical peat forest soil (Malaysia)**	**89.22**	**98.44**	***A. caviae***
			**WS**	
*A. media* WS^a^	Lake (China)	88.55	100	*A. media*
***A. hydrophila* 4AK4**	**Raw sewage (China)**	**88.48**	**93.92**	“***Aeromonas* sp.nov.”**
*A. jandaei* Riv2	River water (USA)	88.61	ND	*A. jandaei*
*A. diversa* 2478-85^T^ ^a^	Leg wound (USA)	84.39	ND	*A. diversa*
*A. molluscorum* 848^T^ ^a^	Bivalve molluscs (Spain)	85.40	ND	*A. molluscorum*
*A. taiwanensis* LMG 24683^T^	Wound (Taiwan)	88.85	ND	*A. taiwanensis*
*A. enteropelogenes* 19991CR	Cerebrospinal fluid (Brazil)	88.17	ND	*A trota* ^e^ *(A. enteropelogenes)*

^a^Genomes also included in the EZ-Genome database up to 23 July 2013 (http://www.ezbiocloud.net/ezgenome/browse_db). Given genomes names at the Ez-Genome for b-e:

^b^ *A. hydrophila*

^c^ *A. veronii*

^d^ *A. cavernicola*.

^e^ *A. enteropelogenes* (a later synonym of *A. trota*). ND, No data can be calculated because no other genome of this species was available.

To determine the consistency of the results obtained with the ANI calculator web-interface in relation to the other two ANI calculating tools available at EzGenome (http://www.ezbiocloud.net/ezgenome/ani) and JSpecies (http://www.imedea.uib.es/jspecies), the three methods were evaluated in parallel with a subset of 15 genomes. Three-independent calculations were performed for each genome comparison using the three tools.

## Results and Discussion

### Determination of taxonomic accuracy among sequenced Aeromonas genomes

**Figure 1:**
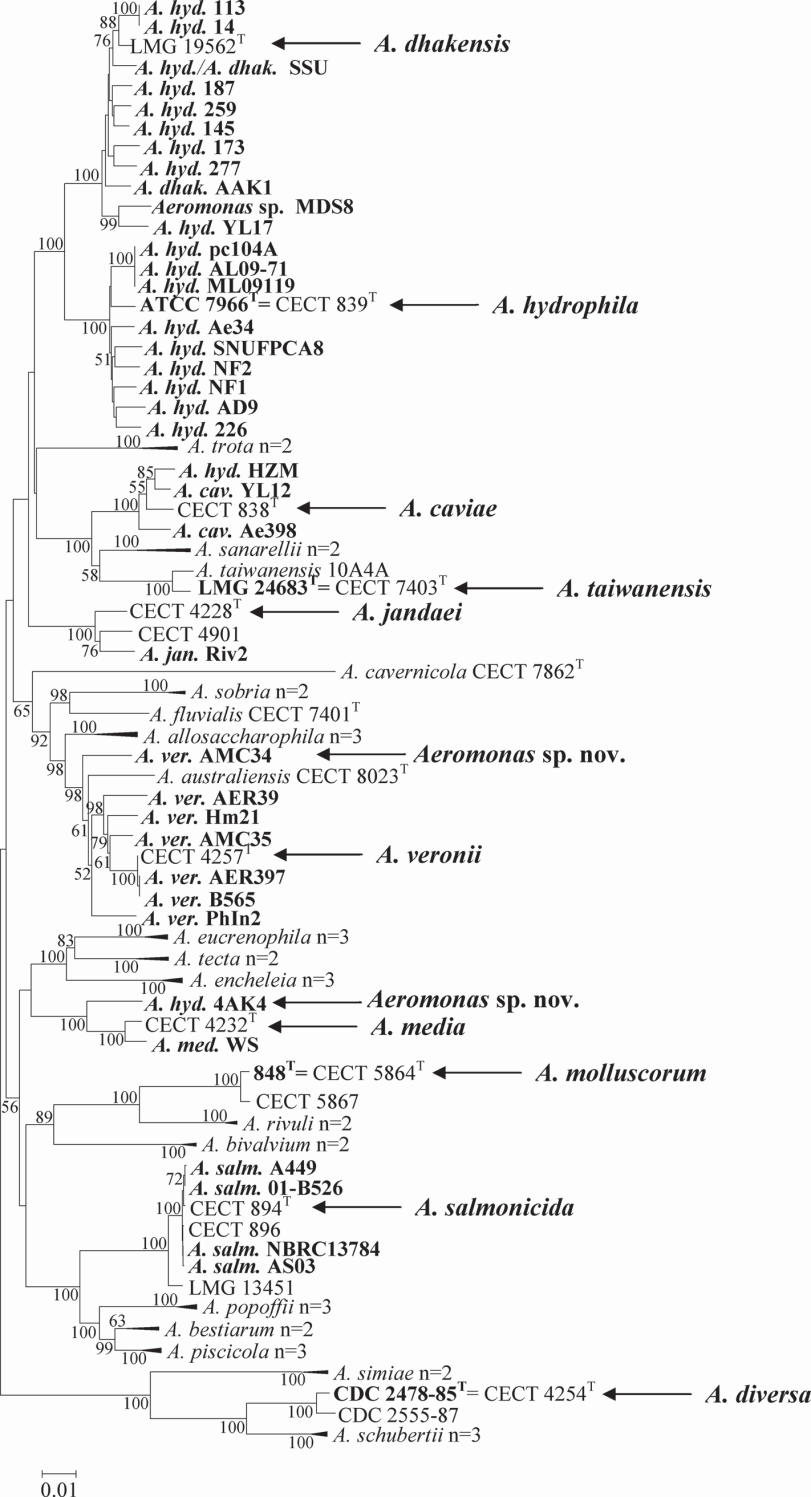
Phylogenetic tree constructed with partial sequences of six concatenated housekeeping genes (*gyrB, rpoD, recA, dnaJ, gyrA, dnaX*, 3879 bp) from 41 *Aeromonas* genomes (in bold as well as their species affiliation) and from 48 strains representing the 27 species included in the genus. Sequences of three genomes (*Aeromonas* sp. 159, *A. enteropelogenes* (*A. trota*) 1991CR and *A. salmonicida* subsp. *salmonicida* 34mel^T^) are not included in the tree as some genes were missing. Abbreviations for the species names: *A. hyd., A. hydrophila; A. dhak., A. dhakensis; A. cav., A.caviae; A. jan., A. jandaei; A. ver., A. veronii; A. med., A. media; A. salm., A. salmonicida*.

As would be expected, the five genomes from *Aeromonas* type strains were correctly labeled as verified by the MLPA (using six concatenated genes *gyrB, rpoD, recA, dnaJ, gyrA, dnaX*, 3879 bp), and ANI results (Fig. 1 and Table 1). This was also verified for the genome of *A. salmonicida* subsp. *salmonicida* 34mel^T^ using the five available concatenated genes (*gyrB, rpoD, recA, dnaJ* and *gyrA*, 3486 bp) (data not shown). A correct phylogenetic affiliation was also observed for 25 (64%) of the remaining 39 *Aeromonas* genomes. These genomes clustered with their corresponding type strains in the MLPA, showed ANI values of > 95% with other strains of their same species (if available) and had ANI values of < 95% with other species (Fig. 1 and Tables 1–3). The same was confirmed for the genome of *A. enteropelogenes* (synonym of *A. trota*) 1991CR in a MLPA with the five available genes (data not shown). In contrast, discordant results were observed for 14 (36%) of the *Aeromonas* genomes in relation to their taxa assignation. Of the 14 that had a listed taxonomic affiliation that was inconsistent with the phylogenetic analyses 12 were misidentified genomes. The remaining two *Aeromonas* sp. genomes (MDS8 and 159), clustered with the type strain of *A. dhakensis* (Fig. 1) and *A. veronii* respectively (using fourconcatenated genes (*gyrB, rpoD, gyrA* and *dnaX*, 2491 bp, data not shown) and showed ANIs > 95% with other representatives of those respective species (Table 1). Of the 12 mislabeled genomes, two corresponded to genomes designated as “*A. veronii* AMC34” and “*A. hydrophila* 4AK4” that on the basis of the ANI and MLPA could belong to two potential new species (Fig. 1 and Tables 1–3).

**Table 2:** ANI results in function of the employed calculating tool for 15 selected *Aeromonas* genomes against a representative one from the same species.

**Reference at NCBI**	**Re-identification MLPA**	**ANI Calculator (ANIb)**	**EzGenome (ANIb)**	**JSpecies (ANIb)**	**JSpecies (ANIm)**
		***A. hydrophila* ATCC7966^T^**			
*A. hydrophila* NF2	*A. hydrophila*	97.29	96.84	96.94	96.76
*A. hydrophila* NF1	*A. hydrophila*	97.15	96.77	96.77	96.66
		***A. dhakensis* SSU**			
*A. dhakensis* AAK1	*A.dhakensis*	97.52	97.12	97.24	97.09
*A. hydrophila* 277	*A. dhakensis*	97.53	97.34	97.30	97.36
*A. hydrophila* 14	*A. dhakensis*	97.54	97.22	97.29	97.09
		***A. veronii* B565**			
*A. veronii* AER39	*A. veronii*	96.76	96.36	96.41	96.52
*A. veronii* Hm21	*A. veronii*	96.83	96.60	96.38	96.38
*A. veronii* Phln2	*A. veronii*	96.75	96.23	96.30	96.48
*Aeromonas* sp. 159	*A. veronii*	96.75	96.26	96.15	96.27
*A. veronii* AMC34	“*Aeromonas* sp. nov.”	94.68	93.51	93.68	94.17
		***A. salmonicida* A449**			
*A. salm.* subsp. *salmonicida* 34mel^T^	*A. salmonicida*	97.40	96.88	96.98	96.98
*A. salm.* subsp. *masoucida* NBRC 13784	*A. salmonicida*	99.82	99.76	99.73	99.51
		***A. caviae* Ae398**			
*A. caviae* YL12	*A. caviae*	98.27	98.12	97.86	97.70
*A. hydrophila* HZM	*A. caviae*	98.44	98.13	98.13	98.07
		***A. media* WS**			
*A. hydrophila* 4AK4	“*Aeromonas* sp. nov.”	93.92	93.10	93.10	93.25

**Table 3:** ANI results in function of the employed calculating tool of 15 selected *Aeromonas* genomes against *A. hydrophila* strain ML09-119.

**Reference at NCBI**	**Re-identification MLPA**	**ANI Calculator (ANIb)**	**EzGenome (ANIb)**	**JSpecies (ANIb)**	**JSpecies (ANIm)**
		**ANI values with *A. hydrophila* ML09-119**			
*A. hydrophila* NF2	*A. hydrophila*	96.94	96.77	96.77	96.53
*A. hydrophila* NF1	*A. hydrophila*	96.79	96.61	96.63	96.05
*A. dhakensis* AAK1	*A. dhakensis*	93.73	92.75	92.76	93.12
*A. hydrophila* 277	*A. dhakensis*	93.76	92.80	92.82	93.13
*A. hydrophila* 14	*A. dhaknesis*	93.77	92.80	92.85	93.22
*A. veronii* AER39	*A. veronii*	88.38	85.29	85.31	87.62
*A. veronii* Hm21	*A. veronii*	88.47	85.29	85.31	87.59
*A. veronii* Phln2	*A. veronii*	88.51	85.58	85.60	87.67
*Aeromonas* sp. 159	*A. veronii*	88.40	85.02	85.04	87.58
*A. veronii* AMC34	“*Aeromonas* sp. nov.”	88.54	85.22	85.23	87.84
*A. salmonicida* subsp. *salmonicida* 34mel^T^	*A. salmonicida*	88.33	86.45	86.43	86.43
*A. salmonicida* subsp. *masoucida* NBRC 13784	*A. salmonicida*	88.35	86.28	86.40	87.78
*A. caviae* YL12	*A. caviae*	89.02	86.18	86.19	88.21
*A. hydrophila* HZM	*A. caviae*	89.22	86.24	86.26	88.25
*A. hydrophila* 4AK4	“*Aeromonas* sp. nov.”	88.48	86.95	87.76	85.85

The majority of the mislabeled genomes were originally designated as *A. hydrophila* (Fig. 1 and Table 1). Only nine out of the 20 *A. hydrophila* genomes (45%) cluster together in the MLPA with the genome of *A. hydrophila* ATCC 7966^T^ and showed ANIs > 95% with the latter and with *A. hydrophila* ML09-119 genome. However, the other 11 “*A. hydrophila*” genomes (55%) showed ANIs < 95% with *A. hydrophila* ML09-119 and cluster in the MLPA with the type strains of other species (Fig. 1 and Table 1). Of these 11 misidentified *Aeromonas* genomes, nine cluster with the type strain of *A. dhakensis* as did genomes, *A. dhakensis* AAK1 and *Aeromonas* sp. MDS8 (Fig. 1). Given the recent taxonomic description of *A. dhakensis* [3], it is perhaps not surprising that the largest proportion of misidentified *Aeromonas* genomes are affiliated with *A. dhakensis*.

One of the misidentified *Aeromonas* genomes was designated as “*A. hydrophila* HZM”, but affiliated in the MLPA with *A. caviae* (Fig. 1). The ANI between “*A. hydrophila* HZM” and *A. caviae* Ae398 was 98.4% and clearly indicated that they belonged to the same species (Tables 1 and 2).

The last example of a misidentified *Aeromonas* genome was “*A. hydrophila* 4AK4”, which did not cluster with *A. hydrophila* but formed a branch close to, but separated from *A. media* in the MLPA (Fig. 1). The genome of “*A. hydrophila* 4AK4” has an ANI value with *A. hydrophila* ML09-119 of 88.5% (Tables 1 and 3), confirming the lack of affiliation with *A. hydrophila*. However, when the wrongly labeled “*A. hydrophila* 4AK4” genome was compared with the only available *A. media* WS genome (that clusters with 100% bootstrap in the MLPA with the type strain of this species) the ANI was low (93.9%) indicating that genome 4AKA could belong to a different species (Table 1 and Fig. 1). This result is in agreement with the data presented in June 2014 at the 11^th^ International Symposium on *Aeromonas* and *Plesiomonas* that indicated that the species *A. media* may contain at least two other species (Talagrand E. et al., personal communication).

The other potential new species among the sequenced *Aeromonas* genomes corresponds to *A. veronii* AMC34 that in the MLPA formed a separate phylogenetic line close to *A. veronii* (Fig. 1). This correlated well with the ANI obtained (94.7%) between AMC34 and the genome of *A. veronii* B565 (Table 1). Both results are in agreement with results of ANI and of a phylogenetic analysis with 16 housekeeping genes presented in a very recent study that included the genome of *A. veronii* AMC34 [6]. Good concordance has also been previously demonstrated between the ANI results and the phylogenetic sequence analysis of some specific genes for several taxa by Konstantinidis et al. [17].

After the re-identification performed in this study the proportion of genomes of the different *Aeromonas* species changed to *A. dhakensis* (11/44, 25%) being the species with the most sequenced genomes followed by *A. hydrophila* (10/44, 22.7%), *A. veronii* (6/44, 13.6%) and *A. caviae* (3/44, 6.8%). In summary, our analysis shows that, excluding the five type strains available in the NCBI, 30.7% (12/39) of the *Aeromonas* genomes were misidentified. This finding is in agreement with the recent study by Colston et al. [6] that also found mislabeled *Aeromonas* genomes in GenBank and suggested that these entries should be corrected to avoid further misidentifications. Furthermore, using the genome information (housekeeping genes) unidentified strains could be identified to the species level and potential new species could be recognized.

### *Aeromonas* ANI inter and intra-species boundaries

A preliminary approach to determine the inter-species ANI variation among the 11 known species represented by 44 *Aeromonas* genomes was the comparison of all of them with the genome of *A. hydrophila* ML09-119. The ANI values generated by comparing the latter genome with others of *A. hydrophila* were in the 96–97% range, supporting their affiliation to *A. hydrophila* (Tables 1 and 3). Closely related species like *A. dhakensis* showed values of 92–93%, while for more distantly related species like *A. veronii* or *A. salmonicida* the pairwise comparison with ML09-119 generated ANIs that were in the range of 84 to 89% (Table 3).

The obtained intra-species range (96.79–99.99%) of ANI values for the 36 genomes of the 5 *Aeromonas* spp. from which more than one genome is available indicated species- specific differences. While this analysis suffers from a relatively small number of available genome sequences, this may indicate to some degree the genomic heterogeneity present among the *Aeromonas* species. Listed in order of the highest intra-species ANI to the lowest, *A. salmonicida* had a range of 97.4% to 99.9% (mean = 99.3%, n = 5), *A. caviae* 98.3% to 98.4% (mean = 98.4%, n = 3), *A. hydrophila* 96.8% to 99.9% (mean = 97.2%, n = 10), *A. dhakensis* 97.3% to 97.6% (mean = 97.5%, n = 11) and *A. veronii* 96.8% to 99.7% (mean = 96.9%, n = 7) (Table 1). It was observed that the species *A. veronii* was the most heterogeneous, having the lowest (96.9%) mean ANI value, and this could be due to the diverse origin of the strains with sequenced genomes that came from clinical and environmental samples from 3 different continents (Table 1).

Results from these 36 genomes representing 5 *Aeromonas* species seems to indicate that a species cutoff ANI value >96% could be the appropriate one for this genus. In fact this is in agreement with results obtained in a recent study that evaluated 56 *Aeromonas* genomes including those of the type strains from 26 of the 27 species of the genus [6]. However, more genomes of closely related species like *A. bestiarum, A. picicola* and *A. salmonicida* or *A. veronii* and *A. allosaccharophila* need to be investigated in order to see if this value should be adjusted. In this sense, as more *Aeromonas* genomes become available in the near future this proposed ANI cutoff value can be confirmed or modified. In agreement with Rodríguez and Konstantinidis [23] the analysis performed in this study shows that ANI offers robust resolution between genomes that share 80–100% ANI.

Taking advantage of the number of existing genomes of *A. hydrophila* and the fact that among them it was the one of the type strain (ATCC 7966^T^) we compared the ANI results obtained for the 9 correctly labeled *A. hydrophila* genomes with the one of the type strain and with the genome of a well characterized strain (ML09-119).

Comparisons with both genomes provided the same ANI identification results (> 96%) as shown in Tables 1–3. Therefore, it can be concluded that once a genome is correctly identified it can be used as a reference for ANI calculations. This is especially relevant when genomes of the type strains are not available, as was the case for six of the 11 species with available genomes at the time this study was performed. This can be applied to other bacterial genera with a scarce number of genomes belonging to type strains in order to avoid mislabeling genomes.

### ANI results in function of the employed tool

The results for ANI pairwise comparisons for 15 representative genomes were compared using the three available ANI calculation tools as shown in Tables 2 and 3. The ANIb values obtained with the JSpecies and the EzGenome tools showed slightly different results in 12 of the 15 intra-species calculations and differences ranged from 0.04 to 0.26% (Tables 2 and 3). Both methods provided consistently lower ANI values than the ANI calculator tool employed in this study. For instance, the intra-species range of differences between the ANI calculator and JSpecies was 0.09 to 0.82%, which was very similar to those (0.06 to 0.82%) found between the ANI calculator and the EzGenome (Tables 2 and 3).

Considering that the JSpecies software enables calculation of both the ANIb and ANIm, these values were also compared for the same 15 genomes and the intra-species values differed in 14 of the 15 values ranging from 0.06 to 0.58% (Tables 2 and 3). The JSpecies web site indicates that both algorithms render nearly identical results at ANI values > 90%. However, our obtained results were different in some cases (Tables 2 and 3).

For the inter-species comparisons, the ANI values obtained with the three methods were different. Again the EzGenome and JSpecies tools provided very similar results differing only from 0.01 to 0.12% but both methods showed higher differences with the ANI calculator that ranged from 0.96 to 3.38% and from 0.92 to 3.36%, respectively (Table 3). For instance for the closely related species *A. hydrophila* and *A. dhakensis* (ANIs between them ranging from 92.8 to 93.8%) the ANI calculator differences in relation to EzGenome (0.96 to 0.98%) and JSpecies (0.92 to 0.97%) were almost 1%. However, the difference between the methods (EzGenome and JSpecies with respect to the ANI calculator) increase even more (1.88 to 3.38% and 1.9 to 3.36% respectively) when more distantly related species were compared, for example *A. hydrophila* ML09-119 compared to *A. veronii, A. salmonicida* or *A. caviae* that showed ANIs ranging from 85.0 to 89.2% (Table 3).

For *Aeromonas* genome comparisons, the three ANI tools provided congruent results with the MLPA analysis. The observed variations among the ANI tools can be relevant like in the case of the genome of “*A. veronii* AMC34” that when compared using the ANI calculator with *A. veronii* B565 gives an ANIb of 94.7% (Table 1, 2) similar to the one obtained with the rest of *A. veronii* genomes (data not shown). This 94.7% ANI value can lead to different interpretations, because it is borderline when considering an ANI species cutoff > 95% or could indicate the same species with a cutoff > 94% in taxa like the genus *Bacillus* [13]. We suggest that “*A. veronii* AMC34” could belong to a potentially new species (ANI < 95%, in agreement with the MLPA). This was further confirmed by the lower ANI values obtained using other ANI tools; the EzGenome (93.51%) and JSpecies (93.7% and 94.2% for ANIb and ANIm, respectively) (Table 2). In these cases the use in parallel of the three methods provided stronger evidence that could help to decide if the evaluated genomes belong or not to a certain species.

In conclusion, the variation observed between the three ANI calculating tools provided intra-species values > 96% and inter-species values < 96% for the compared genomes that did not affect the classification. However, the higher values obtained with the ANI calculator may be critical in borderline situations, where the use in parallel of at least one other ANI calculating tool is highly recommended. In addition, considering that EzGenome and JSpecies provide lower values than the ANI calculator tool, the *Aeromonas* species cutoff value > 96% derived with the latter results should be slightly lowered to embrace the use of any of the methods.

In order to assess the consistency of the 3 ANI calculating tools, 3 independent ANI determinations were performed using each tool. It was observed that the ANI calculator values were identical in the three independent analyses (data not shown). However, using EzGenome we observed that in 25 of the 45 (55.5%) times that the ANI was calculated the values were different within the range 0.01–0.36% (data not shown). In the case of the JSpecies this occurred only in 9 of the 45 (20%) occasions for the ANIb and the range of variation was also low (0.1–0.25%), while with ANIm 13 of the 45 (28.9%) determinations showed different values within the range 0.05–0.44%. These results should alert researches that when using these tools, different ANI values can be obtained; although, these differences do not alter the species classification.

### Genomes names at different databases

In the NCBI GenBank database the genome of strain SSU appears under two different designations, as either *A. hydrophila* (http://www.ncbi.nlm.nih.gov/genome/1422?genome_assembly_id=171265, as of 23 July 2014) or *A. dhakensis* (http://www.ncbi.nlm.nih.gov/genome/genomes/24155, as of 23 July 2014) which causes taxonomic confusion. Strain SSU from which many virulence factors have been discovered was previously known as *A. hydrophila* but was recognized to affiliate with *A. dhakensis* on the basis of ANI comparisons [10] as confirmed also in this study by ANI and MLPA. The curated EzGenome database aims to correct taxonomical errors of the Genome database of the NCBI and includes only 28 genomes that are coincidentally present at the Genome NCBI and the taxonomic name of 15 of them has been changed (http://www.ezbiocloud.net/ezgenome/browse_db). In 11 cases to the subspecies level (9 *A. hydrophila* and 2 *A. salmonicida*), the two *Aeromonas* sp. (159 and MDS8) have been assigned to a species, *A. aquariorum* AAK1 (named correctly at NCBI as *A. dhakensis*) was named as *A. hydrophila* subsp. *hydrophila* and *A. caviae* Ae398 was renamed as *A. cavernicola* (Table 1). However, all the changes except one are erroneous considering the results obtained in this study with the ANI and the MLPA. The only change in agreement with our results is the species name *A. veronii* given to the genome of *Aeromonas* sp. 159. The most striking change is the proposed name of *A. cavernicola* for the genome of *A. veronii* Ae398 when in fact it was originally correctly named at the NCBI as confirmed in this study (Table 1, Fig. 1).

### Impact of mislabeling *Aeromonas* species

The three species (*A. dhakensis, A. caviae* and a potential new species) revealed by the phylogenetic analyses as being misidentified in GenBank as *A. hydrophila* reinforces what has previously been demonstrated in several studies that the incidence of *A. hydrophila* is overestimated using phenotypic methods that masks the real diversity of other *Aeromonas* species [2, 7, 8]. In this study the majority (9/11) of these misidentifications belonged to the recently proposed new species *A. dhakensis* [3] which is in process of publication at the validation list (synonyms are *A. aquariorum* and *A. hydrophila* subsp. *dhakensis*). This species has recently been recognized as an emerging human pathogen being the first or second most prevalent clinical species in Taiwan, Malaysia and Australia [7]. Confusion between *A. hydrophila* and *A. dhakensis* may have clinical consequences considering that the latter species has been found to be more virulent exhibiting more cytotoxicity in human normal skin fibroblast cells than *A. hydrophila* [4]. Furthermore minimum inhibitory concentrations (MICs) of ceftriaxone, imipenem and gentamicin were higher for *A. dhakensis* isolates than for those of *A. hydrophila* [4]. In another study 13 of 16 of *A. dhakensis* blood isolates contained the gene bla_AQU-1_, encoding a class C β-lactamase involved in the resistance of cefotaxime and this was not found in the *A. hydrophila* clinical strains studied in parallel [28]. Therefore, incorrect identification of these two important clinical species can have a negative impact on the evolution and management of the infection. Furthermore, this may hamper the epidemiology of *Aeromonas* infections as the true interspecies variation in the virulence capacities, antimicrobial susceptibility and geographical distribution is masked.

## Conclusions

In an era of rapid and inexpensive genome sequencing, there is less of an impediment to generate genome sequences from a wide diversity of organisms. With the submission of genome sequences to public databases comes the responsibility of assigning the correct taxonomic affiliation. In order to avoid mistakes in labeling genomes, researchers should re-evaluate the strain’s identity once they have the genome and before uploading it to a database. Depending upon how the isolate was originally identified (phenotypically or genetically using 16S rRNA gene similarity) the original given species name may be incorrect. A BLAST comparison using the 16S rRNA or another housekeeping gene is the common practice to search for the correct identification. However, these BLAST analyses can provide misleading results as the same percentage identity can be obtained with different closely related *Aeromonas* species. To avoid mislabeling authors should make an ANI calculation with the type strain if available of the suspected closest species and if it is not available they should use other genomes present in the database under the same species name. This of course becomes problematic if the databases have misleading taxonomic information for sequenced genomes. If the ANI data generated with other related species are > 95% then they should be confident with their results, whereas ANI values below this threshold may indicate a different species. In case of doubt or in absence of a genome sequence a phylogenetic analysis of the concatenated housekeeping gene sequences (using at least two loci, e.g. *gyrB, rpoD* but the more the better) with those of the type strains of closely related species is recommended to ascertain its species identity.

Our results should be an alert for reviewing the 237 entries for *Aeromonas* genomes in the sequence read archives of the NCBI (as of 23 July 2014). Furthermore, the mislabeling found in the curated EzGenome database should prompt a revision of the curating procedure considering that this database indicates that its objective is to correct existing errors at the NCBI. As the number of genome sequences is growing exponentially it is necessary to implement measures to correct and prevent taxonomical errors in public databases to avoid compounding the error by other researchers using these data and reaching incorrect conclusions in comparative genomic studies.

In conclusion, the present study shows that the MLPA and ANI calculations are excellent tools for assigning a genome to the correct species. However, ANI calculations against the type strains will be the fastest and easiest way to assign genomes to the correct taxa.
